# Dr. Hay’s Arctic Expedition

**Published:** 1860-08

**Authors:** 


*Dr. Hays' Arctic Expediton.—The Views of its Commander.*—Dr. Hays, the Commander of the Arctic Expedition, recently gave the following outlines of his views and intentions:

He assured the committee of his perfect satisfaction with the vessel they had purchased. She had been amply strengthened, and was as secure as wood and iron could make her. She was equal, if not superior, to the "Advance," in all the qualities requisite to her peculiar service. All could be done with her that could be done with any vessel. He could not much regret that he had not steam, for the space required for coal bunks and engines would occupy in a steamer so much space that there would not be much room for provisions.

In this vessel there was sufficient food for a three years' cruise, and with economy, for a longer period. In the character and quality of the provisions supplied by the committee, he was thoroughly content. He could not ask for a more perfect outfit. Although he claimed to be a prudent man, if he had found any fault, it would have been that he had too much. All were familiar with the purposes of the expedition, which were to reach the North Pole, and to complete the exploration of the Polar Sea, which was commenced by Dr. Kane. He would push forward with all possible dispatch this summer, and, reaching Smith's Strait early in December, select a harbor as far north as 79 or 80 degrees north latitude, if possible, and there pass the winter. To effect this purpose, he would have some slight advantages in leaving earlier in the season; but during the month of August the ice was more open, and he felt very confident in being able to reach a suitable harbor this season. Next spring he would carry forward provisions, with the aid of dogs; and he would follow in person with the Francis's metallic lifeboat, in which his guests had just come on board.—Should he have the happy fortune to meet open water, then he would launch this boat upon it, and push off for the Pole.

Should they not meet the open sea, they would pass another winter in the ice, and attempt to reach the North Pole by ice alone. He had, however, a firm belief they would meet an open navigable sea.

For the accomplishment of these purposes, every thing, of course, depended upon the vessel which the committee had just presented him. Yet, with such a craft, he felt confident and hopeful. Referring to the inception of the expedition, he said it was true that the first proposal was made by him to the Geographical Society of New York. The contributions of that city and Philadelphia had been liberal and satisfactory; but, owing to the accident of finding a vessel so admirably suited for the service, and the lateness of the season, which precluded the posibility of sending the vessel to New York, the exhibition was now about to sail from Boston. The liberality displayed by the Boston committee, and the citizens of Boston, was unbounded. In his officers and men he had perfect reliance. They were all devoted to the cause. The astronomer of the expedition, Mr. Sontag, whom he had appointed second in command, had been his companion through previous dangers and privations, and having tried him, he knew him to be earnest, brave, and intelligent. The scientific world might well entrust important interest of the expedition to his intelligent zeal.

His executive officer, or master, Captain McCormick, had been known to him for several months, and he had implicit faith in his devotion to the cause, and his determined energy of character. He was a worthy sailor and a brave man.—His second officer was no less devoted, and, although he had known him only for a short period, he was satisfied that he possessed those qualities of coolness, caution, and daring, needed for stern emergencies. He had about him a corps of young, well educated, and intelligent men, who would not only render valuable assistance in the scientific labors of the expedition, but who, with a zealous love of enterprise and adventure, would soon adapt themselves to the narrow quarters, and their calling. The men who went before the mast upon small pay, with no other compensation but their outfit, bore about them those marks of manly courage and endurance, which were calculated to inspire a commander with high hopes of gallant followers. Viewed altogether, he believed that if success did not crown his endeavors, it would not be for the want of any of the means to accomplish his end.

Many misgivings had existed in the minds of persons appealed to by the committee, on the ground of the hazardous nature of the undertaking, and fears had been expressed that an expedition would have to be sent after him. He would briefly say, that he desired no such effort to be made, even should they not return after an absence of two winters, for they had abundant stores, and that he believed that wherever they might be, if alive, they could find their way home, even should their vessel be lost in the ice. Whatever fortune might happen to them, he will keep his crew at the vessel, either until the objects of the expedition were accomplished, or until a further stay in the region becomes unsafe. Then, and not until then, would he return home.—*Medical & Surgical Reporter*.

